# Effectiveness of transcutaneous electrical nerve stimulation for the treatment of migraine: a meta-analysis of randomized controlled trials

**DOI:** 10.1186/s10194-018-0868-9

**Published:** 2018-05-29

**Authors:** Huimin Tao, Teng Wang, Xin Dong, Qi Guo, Huan Xu, Qi Wan

**Affiliations:** Department of Neurology, The First Affiliated Hospital of Nanjing Medical University, 300 Guangzhou Road, Nanjing, 210029 Jiangsu Province China

**Keywords:** Migraine, Transcutaneous electrical nerve stimulation, TENS, Meta-analysis

## Abstract

**Background:**

Migraine is now ranked as the second most disabling disorder worldwide reported by the Global Burden of Disease Study 2016. As a noninvasive neurostimulation technique, transcutaneous electrical nerve stimulation(TENS) has been applied as an abortive and prophylactic treatment for migraine recently. We conduct this meta-analysis to analyze the effectiveness and safety of TENS on migraineurs.

**Methods:**

We searched Medline (via PubMed), Embase, the Cochrane Library and the Cochrane Central Register of Controlled Trials to identify randomized controlled trials, which compared the effect of TENS with sham TENS on migraineurs. Data were extracted and methodological quality assessed independently by two reviewers. Change in the number of monthly headache days, responder rate, painkiller intake, adverse events and satisfaction were extracted as outcome.

**Results:**

Four studies were included in the quantitative analysis with 161 migraine patients in real TENS group and 115 in sham TENS group. We found significant reduction of monthly headache days (SMD: -0.48; 95% CI: -0.73 to − 0.23; *P* < 0.001) and painkiller intake (SMD: -0.78; 95% CI: -1.14 to − 0.42; P < 0.001). Responder rate (RR: 4.05; 95% CI: 2.06 to 7.97; P < 0.001) and satisfaction (RR: 1.85; 95% CI: 1.31 to 2,61; P < 0.001) were significantly increased compared with sham TENS.

**Conclusion:**

This meta-analysis suggests that TENS may serve as an effective and well-tolerated alternative for migraineurs. However, low quality of evidence prevents us from reaching definitive conclusions. Future well-designed RCTs are necessary to confirm and update the findings of this analysis.

**Systematic review registration:**

Our PROSPERO protocol registration number: CRD42018085984. Registered 30 January 2018.

**Electronic supplementary material:**

The online version of this article (10.1186/s10194-018-0868-9) contains supplementary material, which is available to authorized users.

## Background

Migraine is now ranked as the second most disabling disorder worldwide reported by the Global Burden of Disease Study 2016 [1], which is characterized by recurrent moderate to severe unilateral throbbing head pain accompanied by photophobia, phonophobia, nausea and vomiting [2]. Therapeutic strategies are mainly based on both preventive and abortive drug therapy. However, conventional pharmacological therapies are partially effective and have unpleasant adverse effects inevitably. Overuse of symptomatic medication for headaches may lead to drug resistance and even transformation into refractory medication overuse headache [3]. Therefore, nonpharmacological therapeutic strategies with better efficacy and tolerance are pressingly needed.

Transcutaneous electrical nerve stimulation (TENS) is the delivery of pulsed low voltage electrical currents across the intact surface of the skin to stimulate peripheral nerves principally for pain relief [4]. As a noninvasive neurostimulation technique, TENS has gradually been the subject of extensive research in the treatment of headache disorders. Cefaly® is the first medical device approved by the FDA as a prophylactic treatment for episodic migraine, which stimulates the supratrochlear and supraorbital nerves [5]. Another novel non-invasive transcutaneous vagal nerve stimulation device, nVNS gammaCore, has been developed and is CE marked for acute and prophylactic treatment of primary headache disorders including cluster headache and migraine [6–8].

Although several clinical trials applying TENS as an abortive or prophylactic treatment for migraine have been carried out, there is no rigorous systematic review, to the best of our knowledge, investigating the effectiveness and safety of TENS in migraineurs. Therefore, the aim of this meta-analysis was to assess the evidence from randomized controlled clinical trials that used TENS for pain relief in migraine patients.

## Methods

This meta-analysis was conducted according to the guidance of the Preferred Reporting Items for Systematic Reviews and Meta-analysis statement [9]. The review protocol was registered in the International Prospective Register of Systematic Reviews and the registration number was CRD42018085984.

### Eligibility criteria

Studies were identified based on the following criteria: (1) participants over 18 years old diagnosed with migraine according to the International Classification of Headache Disorders (ICHD-II or ICHD-III beta version); (2) comparing real TENS with sham TENS; (3) reporting migraine days, headache days, migraine attacks, pain intensity, painkiller intakes, adverse events or satisfaction as outcomes; (4) randomized controlled trials.

The exclusion criteria were as follows: (1) comparison with other therapies such as drugs or psychotherapy; (2) applying invasive electrical nerve stimulation; (3) other types of trials such as cross-over designs, self-contrast trials and healthy controlled trials.

### Literature search and study selection

Two reviewers (Tao and Wang) independently searched the following electronic databases up to December 2017: MEDLINE (via PubMed), Embase, the Cochrane Library and the Cochrane Central Register of Controlled Trials without language restrictions. The search strategies used can be found in Additional file 1. To avoid omitting relevant trials, conference abstracts and reference lists of all identified related publications were also searched. The computer search was supplemented with manual searches of the reference to expand potentially relevant articles. When multiple reports describing the same population were published, the most complete report was included.

### Data extraction and outcome measures

Data extraction was performed independently by two authors. The following information was extracted from the included RCTs: first author; publication year; country; study design; sample size; study population (age range, gender split, baseline characteristics); intervention (stimulation site, parameters and duration of stimulation); adverse events and outcomes. We contacted to the corresponding authors when the related data were incomplete. Those who did not reply to our data request were excluded from the meta-analysis.

The primary outcomes included changes in monthly headache days between real and sham TENS, evaluated by headache diaries. Percentage of ‘responders’, i.e., of subjects having at least 50% reduction of monthly migraine days between the run-in period and the end of treatment was also investigated as primary outcome measures. Secondary outcomes were painkiller intake, satisfaction and adverse events during or after stimulation.

### Assessment of risk of bias

Risk of bias assessment was performed independently by two authors (Tao and Wang) and adjudicated by a third investigator (Dong) in the event of disagreement, according to Cochrane Collaboration’s tool for assessing bias in randomized trials [10]**.** The domains assessed were sequence generation (selection bias), allocation sequence concealment (selection bias), blinding of participants and personnel (performance bias), blinding of outcome assessment (detection bias), incomplete outcome data (attrition bias), selective outcome reporting (reporting bias) and other potential sources of bias.

### Data analysis

The data synthesis was performed by Review Manager 5.3 (Cochrane Collaboration, Oxford, UK). The standardized mean difference (SMD) and relative risk (RR) were used to compare continuous and dichotomous variables, respectively. All results were reported with 95% confidence intervals (CIs). For studies that presented continuous data as means and range values, the standard deviations were calculated based on the principles of the Cochrane Handbook for Systematic Reviews of Interventions [11].

Heterogeneity was tested using the chi-square test (*P* < 0.1) and quantified with the I^2^ statistic, which described the variation of effect size that was attributable to heterogeneity across studies [12]. I^2^ values smaller than 50% indicate no significant heterogeneity and are acceptable. The fixed-effect model of analysis is the appropriate. Otherwise, the random-effect model is considered.

Prespecified subgroup analysis was performed according to migraine attack frequency (episodic or chronic). Sensitivity analysis was also performed to determine effect size when low-quality studies were excluded. Owing to the limited number (*n* < 10) of included studies, publication bias was not assessed.

Finally, we assessed the quality of evidence by GRADE profiler, considering risk of bias, inconsistency, indirectness, imprecision, and publication bias [13].

## Results

### Study selection and inclusion

**Fig. 1:**
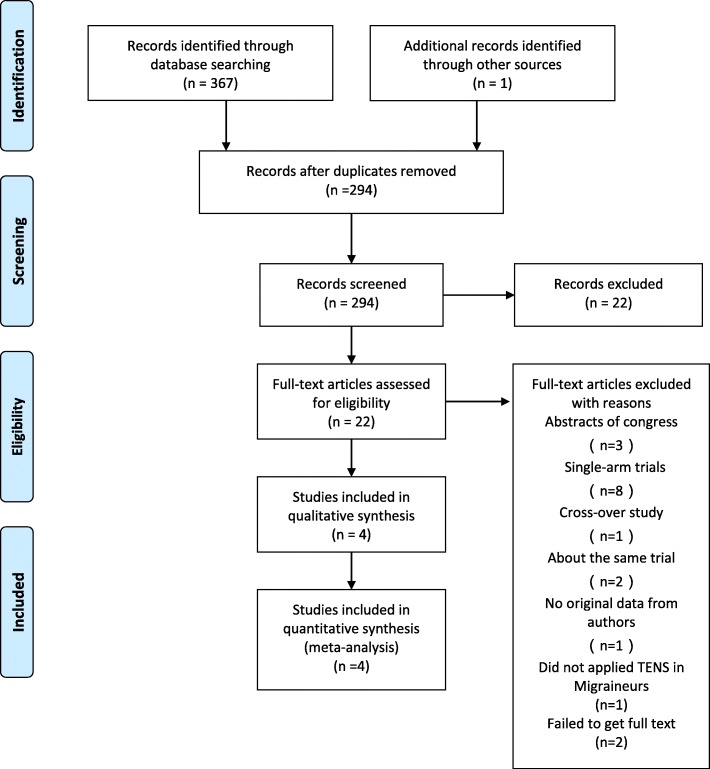
Flow diagram of studies. Process of identifying eligible studies for the meta-analysis

The flow chart for the selection process and detailed identification was presented in Fig. 1. Search strategies identified 368 potentially relevant publications. After the removal of duplicates, 294 articles were spotted, but only 22 remained after screening titles and abstracts. In the eligible articles, one trial enrolled both tension-type headache patients and migraineurs [14], and we were unable to extract data of migraineurs separately. We failed to contact the authors for the detail data until the end of this review. Ultimately, four RCTs, enrolling a total of 276 patients were included in the meta-analysis [15–18].

### Study characteristics

The characteristics of the studies included are summarized in Table 1. The four studies were published between 2013 and 2017 in English. Two of them were multicenter trials in Belgium [15] and the USA [16], the others were monocenter trials in China [17, 18]. Patients with at least 2 migraine attacks each month or chronic migraine were recruited in the trials. The four included studies ranged in size from 59 to 88 subjects and from 1 to 8 months in duration. Different TENS manufacturers applied pulsed electrical stimulation to supraorbital nerves (the branch of the trigeminal nerve), vagus nerves, occipital nerves and Taiyang (EX-HN 5) acupoints (trigeminal nerve indirectly) respectively. Parameters including frequency and amplitude were different among the trials in real TENS groups. In three studies [16–18], the sham group had the same device applied but received no electrical stimulation. In the one study [15], the intensity of sham simulation was far less than the real group. The outcome measurement methods were common across all studies, using headache diaries. One study had a high dropout rate [16] and all studies had an intention-to-treat analysis.

**Table 1:** Characteristics of included studies

Included studies	Country	Groups (n)	Gender female/male	Age	Headache days during baseline	Stimulation location	Parameters	Device/manufacturer	Duration and frequency	Adverse events	ITT	Outcomes (Assessment Tool)
Li et al. 2017	China	EG = 31 CG = 31	EG 28/3 CG 2/29	EG = 35.9 ± 10.6 CG = 37.1 ± 11.4	EG = 7.6 ± 3.7 CG = 7.2 ± 3.3	Bilateral Taiyang acupoints. (trigeminal nerve indirectly)	EG: frequency 2/100 Hz; CG: deliver no electrical stimulation	LH202H Han Electrostimulator, Jinghua Wei Industry Development Company, Beijing, China	5 times weekly for 12 weeks	No	Yes	Change in monthly migraine days, migraine attacks, headache days and acute antimigraine drug intake between run-in and third month of treatment
Schoenen et al. 2013	Belgium	EG = 34 CG = 33	EG 31/3 CG 30/3	EG = 34.6 ± 11.0 CG = 39.1 ± 9.9	EG = 7.8 ± 4.0 CG = 6.7 ± 2.6	Bilateral supratrochlear and supraorbital nerves	EG: biphasic rectangular impulses, pulse width 250 μs, frequency 60 Hz, intensity 16 mA; CG: pulse width 30 μs, frequency 1 Hz, intensity 1 mA	Cefaly, STX-Med., Herstal, Belgium	20 min daily for 3 months	No	Yes	Change in monthly migraine days, 50%response rate, change in monthly migraine attacks, headache days, mean headache severity per migraine day, acute antimigraine drug use between run-in and third month of treatment; satisfaction
Silberstein et al. 2016	USA	EG = 30 CG = 29	EG 26/4 CG 27/2	EG = 40.5 ± 14.2 CG = 38.8 ± 11.1	EG = 20.8 ± 5.0 CG = 22.3 ± 4.9	Vagus nerve	EG: voltage peak 24 V, maximum current of 60 mA; CG: deliver no electrical stimulation	GammaCore®, electroCore, LLC, Basking Ridge, NJ	Two 2-min 3 times a day for 8 months	EG: 12 AEs CG: 8 AEs	Yes	Safety and tolerability, Change of headache days per 28 days, 75% response rate, 50%response rate, acute medication use
Liu et al. 2017	China	EG = 66 CG = 22	EG 52/14 CG 18/4	EG = 37.6 ± 10.4 CG = 44.3 ± 8.3	EG = 11.5 ± 7.2 CG = 9.9 ± 3.9	Bilateral occipital nerves	EG: intensity 10 mA Group A 2 Hz, Group B 100 Hz, Group C 2/100 Hz CG: deliver no electrical stimulation	HANS-200A machine, JiSheng Medical Technology Limited Company, China	30 min daily for 1 month	EG: Group A 1 AE	Yes	50% responder rate, changes in headache days monthly, headache intensity (measured using the VAS), headache duration, scores on SDS、SAS、HIT-6, percentage of satisfaction

*EG* experimental group, *CG* control group, *ITT* intension-to-treat, *AE* adverse event

### Risk of bias

**Fig. 2:**
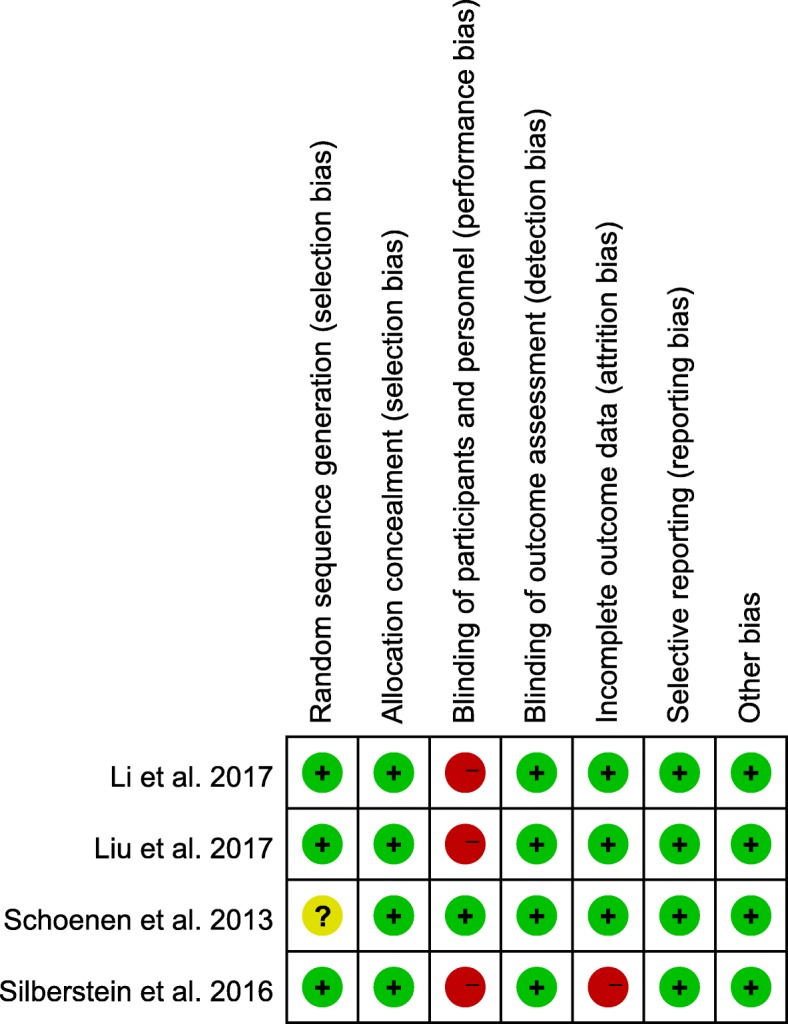
Risk of bias summery for included trials

Figure 2 summarized the risk of bias of four selected studies considering main outcomes. For the criteria sequence generation, we judged one trial as having an uncertain risk of bias [15], because it didn’t provide sufficient information about randomization. All studies reported allocation concealment, therefore, we judged these studies as having low bias. It was noteworthy that, although all the studies claimed to be double-blind trials, it is difficult for patients to achieve a true blindness. For the sham protocol, three studies delivered no stimulation to devices [16–18], thus establishing blinding of participants is difficult. Only in one study both stimulators buzzed identically during treatment [15], and thus it was not possible to distinguish a sham from a real stimulator without testing both devices in parallel. Therefore, we deemed it at low risk of bias and the other three studies at a high risk of bias with respect to blinding of participants. All four studies used the headache diary to evaluate pain control, hence, evaluators could not influence this outcome measure. Therefore, we consider the studies as low risk of with regard to detection bias. One study had high dropout rate and we judge it as having a high risk of bias in terms of incomplete outcome data [16]. All studies utilized intention-to-treat analyses. Reporting bias and other potential sources of bias were judged as low in all included studies.

### Primary outcomes

#### Change in the number of monthly headache days

**Fig. 3:**
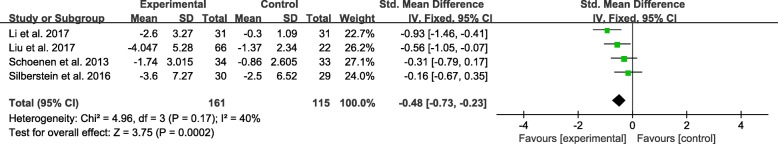
Change in the number of monthly headache days. Forest plot of the meta-analysis showed a significant decrease in the number of monthly headache days after therapy with TENS compared with sham TENS

**Fig. 4:**
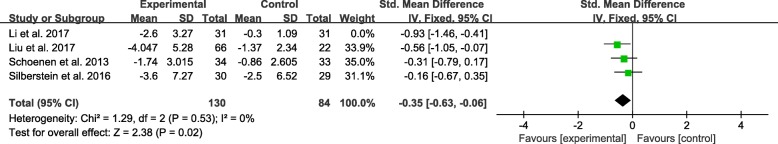
Change in the number of monthly headache days (sensitivity analysis). Sensitivity analysis showed that heterogeneity was most likely because of the study by Li et al., without which the heterogeneity reduced to zero with little change to the summary estimate

The outcome data was analyzed with a fixed-effect model, and the pooled estimate of the four included RCTs suggested that compared with placebo group in migraine patients, real TENS was found to significantly reduce the number of monthly headache days (SMD: -0.48; 95% CI: -0.73 to − 0.23; *P* < 0.001), with moderate heterogeneity among the studies (I^2^ = 40%) (Fig. 3). Sensitivity analysis showed that heterogeneity was most likely because of the study by Li et al. [18], without which the heterogeneity reduced to zero with little change to the summary estimate (Fig. 4). The heterogeneity might be caused by the intervention. In the trial by Li et al. [18], percutaneous electrical nerve stimulation therapy utilized acupuncture-like needle probes insertion into the soft tissues to stimulate trigeminal nerves instead of electrodes.

### Responder rate

**Fig. 5:**
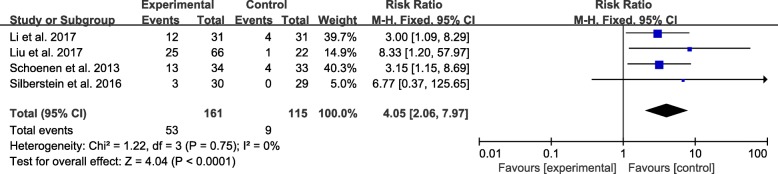
Responder rate. Forest plot of the meta-analysis showed significant increase in 50% responder rate after therapy with TENS compared with sham TENS

All four studies with a total of 276 patients reported the number of responders. Responder rate was significantly higher in real TENS group than in sham TENS group (32.9% and 7.8%; RR: 4.05; 95% CI: 2.06–7.97; *P* < 0.001) (Fig. 5). Furthermore, the meta-analysis result of the included trials found a low level of heterogeneity (I^2^ = 0). Thus, we did not perform sensitivity analysis.

### Secondary outcomes

#### Painkiller intake

**Fig. 6:**
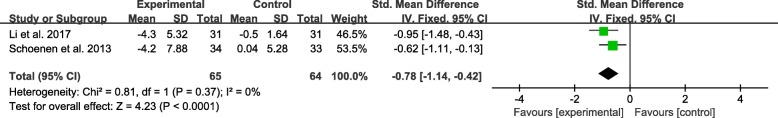
Painkiller intake. Forest plot of the meta-analysis showed a significant decrease in the number of painkiller intake after therapy with TENS compared with sham TENS

Only two studies included reported painkiller intake as an outcome [15, 18]. The pooled estimate of two included RCTs suggested that compared with sham TENS in migraine patients, real TENS yielded significantly decreased monthly painkiller intake (SMD: -0.78; 95% CI: -1.14 to − 0.42; P < 0.001), presented in Fig. 6.

### Adverse events

All studies included mentioned adverse events or side effects related to TENS or sham TENS therapy during the trials. Only one study aimed to assess the feasibility, safety, and tolerability of TENS and reported adverse events in detail [16]. The tolerability profile of noninvasive vagus nerve stimulation (nVNS) was satisfactory and generally similar to that of sham treatment. Most adverse events were mild or moderate and transient. The most commonly reported adverse events were upper respiratory tract infections, facial pain and gastrointestinal symptoms. Two studies explicitly reported no adverse events associated with TENS treatment [15, 18]. In the other study [17], only one patient reported one adverse event in the 2 Hz group. It was a form of pinch pain and the uncomfortable feeling subsided when the intensity of the stimulation was reduced.

### Satisfaction

**Fig. 7:**
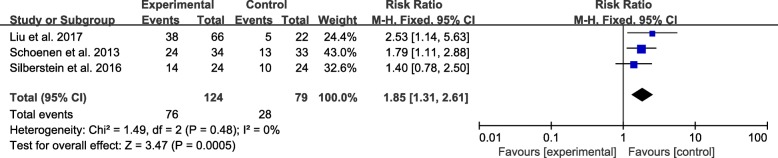
Satisfaction. Forest plot of the meta-analysis showed a significant increase in satisfaction after therapy with TENS compared with sham TENS

Three studies reported the number of people satisfied with the TENS treatment [15–17]. Compared with sham TENS in migraine patients, real TENS yielded significant satisfaction rate. The pooled data of the 104 patients in these three studies showed significantly higher satisfaction rate in the real TENS group than the sham group (RR:1.85; 95% CI: 1.31 to 2.61; *P* < 0.001), with no heterogenicity (I^2^ = 0%) across the studies (Fig. 7).

### GRADE analysis

**Fig. 8:**
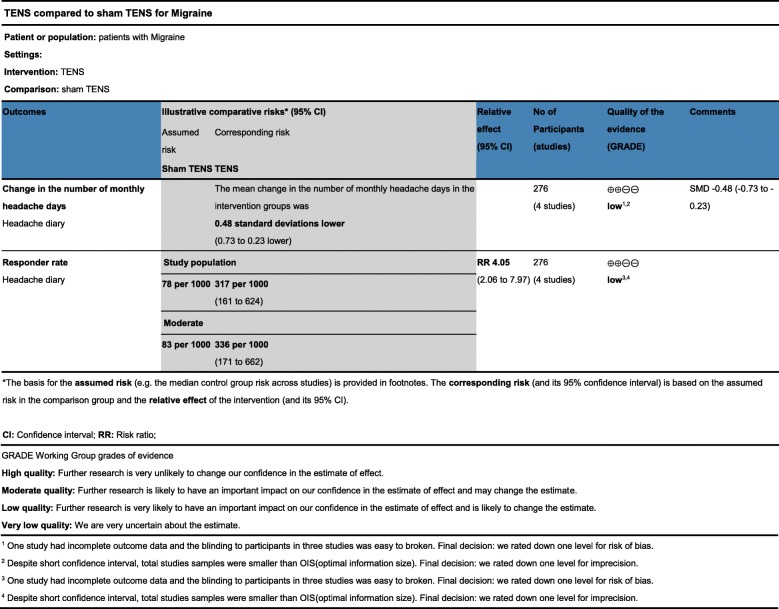
Quality of evidence assessment. Quality of evidence assessment for pain control outcomes performed by GRADE profiler

The quality of evidence for outcomes evaluated in this review was assessed according to GRADE guidelines (Fig. 8) For the outcome of change in monthly headache days, the evidence quality was rated as low. We rated down one level for risk of bias. As samples size was smaller than optimal information size, the quality of evidence was downgraded once again for imprecision. The prevalence of small studies increases the risk of publication bias. There is a propensity for small negative studies not to reach full publication, and this might lead to an exaggerated estimate of effect [19]. We found that some of the trials were registered on clinicaltrials.gov, but the results were not updated in time. However, we did not downgrade for the publication bias as we had no direct evidence of this. For the outcome of responder rate, the evidence quality was rated as ‘low’ similar to change in monthly headache days.

## Discussion

This meta-analysis of 4 RCTs including 276 patients provides evidence that TENS could be an effective and well-tolerated technique in increasing responder rate, reducing headache days and painkiller intake when compared with sham treatment. All the enrolled patients in the included studies didn’t use prophylaxis drugs during the treatment and for 1 month prior to the treatment, which reduced the interference of prophylaxis drugs to a certain degree. However, the quality of the evidence was judged as ‘low’ GRADE due to the methodological limitations of the included studies, and overall small study sizes. Further research is very likely to have an important impact on our confidence in the estimate of effect and is likely to change the estimate.

This is the first meta-analysis, to the best of our knowledge, to investigate the effectiveness and safety of TENS for the treatment of migraine. This result is similar to a 2017 Cochrane review by Gibson et al. [20] and a 2015 Cochrane review by Johnson et al. [4], which were unable to makes definitive conclusions of TENS for acute and neuropathic pain largely because of inadequate sample sizes and unsuccessful blinding of treatment interventions in the included studies.

TENS induced analgesia is thought to be multifactorial and the ‘gate control theory’ is in fact the most conceivable view [21]. Neurostimulation may work by activating large fiber sensory afferents, which may secondarily inhibit nociceptive inputs from small fibers and elevate pain thresholds. Moreover, central descending pain inhibitory systems may be engaged as demonstrated by both animal studies and functional imaging studies [22]. GammaCore may reduce pain through restoration of brainstem monoaminergic neurotransmission [23], suppression of glutamate levels and cortical spreading depression [24, 25]. Cefaly may exert beneficial effects via normalization of orbitofrontal and rostral anterior cingulate cortices hypometabolism [26]. Occipital neurostimulation may active Aβ fibers of trigeminocervical complex in the neck in order to inhibit the pain transmission [17] and restore central descending pain modulatory tone at the same time [22]. Electrical stimulation to Taiyang acupoints, which indirectly stimulates the branch of the trigeminal nerve, improves the endogenous morphine like substance and serotonin in the central nervous system to relieve pain [27, 28]. Despite their unique mechanisms, all stimulated are peripheral nerves, and they have a common basic theory-- ‘gate control theory’. In the future, with the increase in the number of studies, subgroup analysis can be performed according to the type of stimulated nerves to reduce heterogeneity to some extent.

Maintaining blinding is a major methodological challenge in studying TENS. Various types of sham TENS have been proposed including units that are identical in appearance but just deliver an initial brief period of stimulation at the start and then faded out [29]. In some studies, sham stimulation parameters are set below levels needed for therapeutic or even no current is delivered in control group [16–18]. However, active stimulation elicits strong sensations and a true sham treatment that establishes robust blinding of participants is challenge [30].

TENS technique has been applied for both acute treatment of migraine [31, 32] and migraine prevention [15, 17]. Even though some single-arm trials demonstrate the effectiveness of TENS for migraine [8, 31–35], the reliability is downgraded considering the placebo effect. Given that sham TENS methodologies may be inherently flawed, further studies can focus on assessing TENS versus preventive anti-migraine drugs, botulinum neurotoxin, or other nonpharmacologic treatments like neurofeedback and transcranial magnetic stimulation. Assessing conventional therapy versus conventional therapy plus active TENS can also be taken into consideration.

### Limitations

Several limitations should be taken into account. Firstly, our analysis was based on only four RCTs and all of them had a relatively small sample size (*n* < 100). One trial enrolled both tension-type headache patients and migraineurs, and we failed to contact the authors for the detail data until the review was completed. The included studies varied in the number of sessions, stimulation parameters and stimulated nerve types particularly, which increased the potential biases in the studies. Secondly, no subgroup analysis was performed based on the stimulated nerve types owing to the small number of studies included. Thirdly, since only two trials reported headache intensity as outcomes and they differed in measuring method, classified as mild, moderate, severe pain and visual analogue (VAS) scale respectively, thus we didn’t perform a pooled analysis. Finally, the follow-up period was generally short, so long-term outcomes of TENS remain to be proved.

## Conclusions

This meta-analysis indicates that TENS may be effective in increasing responder rate, reducing headache days and painkiller intake, serving as a well-tolerated alternative for migraineurs. Nevertheless, despite our rigorous methodology, the inherent limitations of included studies make it impossible for us to draw definitive conclusions. Blinding of participants should be emphasized in future TENS trials to explore the efficacy of TENS as a sole or adjuvant therapy in patients with migraine, especially suffering from refractory migraine. TENS could be of help also in patients with (or at risk for) medication overuse and in fragile migraine populations, namely children, adolescents, pregnants and elderly. Future large-scale, well-designed RCTs with extensive follow-up are necessary to provide evidence-based efficacy data, optimize our knowledge concerning patient selection, stimulation parameters and update the findings of this analysis.

### Clinical implications

This is the first meta-analysis investigating the effectiveness and safety of TENS for the treatment of migraine.

There is low quality evidence suggesting that TENS may be effective in increasing responder rate, reducing headache days and painkiller intake, serving as a well-tolerated alternative for migraineurs.

Future well-designed RCTs with extensive follow-up are necessary to provide evidence-based efficacy data, optimize our knowledge concerning patient selection and stimulation parameters.

## Additional file

Additional file 1: Search strategy. (DOCX 14 kb)

## Abbreviations

AE: Adverse event
CG: Control group
CI: Confidence interval
EG: Experimental group
ICHD: the International classification of headache disorders
ITT: Intension-to-treat
M-H: Mantel-Haenszel
TENS: Transcutaneous electrical nerve stimulation
