# Supplementary material for: Effectiveness of transcutaneous electrical nerve stimulation for the treatment of migraine: a meta-analysis of randomized controlled trials
Source: J Headache Pain. 2018 May 29;19(1):42. doi: 10.1186/s10194-018-0868-9 (PMC5975046; doi:10.1186/s10194-018-0868-9)
Supplement: Supplementary file 1 — Search strategy. (DOCX 14 kb) [file 10194_2018_868_MOESM1_ESM.docx]

Embase

#1 'transcutaneous electrical nerve stimulation'/exp OR 'transcutaneous electrical nerve stimulation'

#2 'electrostimulation, transcutaneous':ab,ti OR 'nerve stimulation, transcutaneous':ab,ti OR 'percutaneous electric nerve stimulation':ab,ti OR 'percutaneous electrical nerve stimulation':ab,ti OR 'tens (transcutaneous electrical nerve stimulation)':ab,ti OR 'transcutaneous electric nerve stimulation':ab,ti OR 'transcutaneous electrical stimulation':ab,ti OR 'transcutaneous electrostimulation':ab,ti OR 'transcutaneous nerve stimulation':ab,ti

#3 'electroanalgesia':ab,ti OR 'peripheral nerves stimulaion':ab,ti OR 'supraorbital nerve stimulation':ab,ti OR 'vagus nerve stimulation':ab,ti OR 'occipital nerve stimulation':ab,ti OR 'cefaly':ab,ti OR 'gammacore':ab,ti OR 'transcutaneous nerve stimulation':ab,ti

#4  #1 OR #2 OR #3

#5 'headache'/exp OR 'headache'

#6 'migraine'/exp OR 'migraine'

#7 'headache*':ab,ti OR 'migrain*':ab,ti OR 'cephalgi*':ab,ti OR 'cephalalgi*':ab,ti

#8 #5 OR #6 OR #7

#9 'randomized controlled trial':ab,ti OR 'random':ab,ti OR 'placebo':ab,ti OR 'double-blind':ab,ti

#10  #4 AND #8 AND #9
